# Phenotypic characterization of *Leishmania* spp. causing cutaneous leishmaniasis in the lower Amazon region, western Pará state, Brazil, reveals a putative hybrid parasite, *Leishmania* (*Viannia*) *guyanensis* × *Leishmania* (*Viannia*) *shawi shawi*

**DOI:** 10.1051/parasite/2014039

**Published:** 2014-08-04

**Authors:** Yara Lins Jennings, Adelson Alcimar Almeida de Souza, Edna Aoba Ishikawa, Jeffrey Shaw, Ralph Lainson, Fernando Silveira

**Affiliations:** 1 Parasitology Department, Institute Evandro Chagas (Surveillance Secretary of Health, Ministry of Health) Belém Pará state Brazil; 2 Tropical Medicine Nucleus, Federal University of Pará Belém Pará state Brazil; 3 Biomedical Sciences Institute, São Paulo University São Paulo São Paulo state Brazil

**Keywords:** Phenotypic characterization, *Leishmania* spp., Monoclonal antibodies, Isoenzyme electrophoresis, Cutaneous leishmaniasis, Amazonian Brazil

## Abstract

We phenotypically characterized 43 leishmanial parasites from cutaneous leishmaniasis by isoenzyme electrophoresis and the indirect immunofluorescence antibody test (23 McAbs). Identifications revealed 11 (25.6%) strains of *Leishmania* (*V*.) *braziliensis*, 4 (9.3%) of *L*. (*V*.) *shawi shawi*, 7 (16.3%) of *L*. (*V*.) *shawi santarensis*, 6 (13.9%) of *L*. (*V*.) *guyanensis* and *L*. (*V*.) *lainsoni*, 2 (4.7%) of *L*. (*L*.) *amazonensis*, and 7 (16.3%) of a putative hybrid parasite, *L*. (*V*.) *guyanensis*/*L*. (*V*.) *shawi shawi*. McAbs detected three different serodemes of *L*. (*V*.) *braziliensis*: I-7, II-1, and III-3 strains. Among the strains of *L*. (*V*.) *shawi* we identified two populations: one (7 strains) expressing the B19 epitope that was previously considered to be species-specific for *L*. (*V*.) *guyanensis*. We have given this population sub-specific rank, naming it *L*. (*V*.) *s*. *santarensis*. The other one (4 strains) did not express the B19 epitope like the *L*. (*V*.) *shawi* reference strain, which we now designate as *L*. (*V*.) *s*. *shawi*. For the first time in the eastern Brazilian Amazon we register a putative hybrid parasite (7 strains), *L*. (*V*.) *guyanensis*/*L*. (*V*.) *s*. *shawi*, characterized by a new 6PGDH three-band profile at the level of *L*. (*V*.) *guyanensis*. Its PGM profile, however, was very similar to that of *L*. (*V*.) *s*. *shawi*. These results suggest that the lower Amazon region – western Pará state, Brazil, represents a biome where *L*. (*V*.) *guyanensis* and *L*. (*V*.) *s*. *shawi* exchange genetic information.

## Abstract (fr)

Nous avons caractérisé phénotypiquement 43 leishmanies causant la leishmaniose cutanée par électrophorèse des isoenzymes et test d’anticorps par immunofluorescence indirecte (23 McAbs). Les identifications ont révélé 11 (25,6 %) souches de *Leishmania* (*V*.) *braziliensis*, 4 (9,3 %) de *L*. (*V*.) *shawi shawi*, 7 (16,3 %) de *L*. (*V*.) *shawi santarensis*, 6 (13,9 %) de *L*. (*V*.) *guyanensis* et *L*. (*V*.) *lainsoni*, 2 (4,7 %) de *L*. (*L*.) *amazonensis* et 7 (16,3 %) d’un parasite hybride putatif, *L*. (*V*.) *guyanensis × L*. (*V*.) *shawi shawi*. Les McAbs ont détecté 3 sérodèmes différentes de *L*. (*V*.) *braziliensis* : I-7, II-1 et III-3 souches. Parmi les souches de *L*. (*V*.) *shawi* nous avons identifié deux populations : une (7 souches) exprime l’épitope B19 qui était auparavant considéré comme spécifique de l’espèce *L*. (*V*.) *guyanensis*. Nous avons attribué à cette population le rang subspécifique, la désignant comme *L*. (*V*.) *s*. *santarensis*. L’autre (4 souches) n’a pas exprimé l’épitope B19 comme la souche de référence *L*. (*V*.) *shawi* que nous désignons maintenant comme *L*. (*V*.) *s*. *shawi*. Pour la première fois dans l’est de l’Amazonie brésilienne, nous rapportons un parasite hybride putatif (7 souches), *L*. (*V*.) *guyanensis × L*. (*V*.) *s*. *shawi*, caractérisé par un nouveau profil 6PGDH à trois bandes au niveau de *L*. (*V*.) *guyanensis*. Son profil PGM, cependant, est très similaire à celui de *L*. (*V*.) *s*. *shawi*. Ces résultats suggèrent que la région de l’Amazone inférieure -- ouest de l’État du Pará, Brésil, représente un biome où *L*. (*V*.) *guyanensis* et *L*. (*V*.) *s*. *shawi* échangent de l’information génétique.

## Introduction

American cutaneous leishmaniasis (ACL) is a parasitic protozoal disease widespread in most countries of Latin America, and is caused by a variety of *Leishmania* spp. within the subgenera *Viannia* and *Leishmania* [22, 24]. In Amazonian Brazil, there are seven well-known *Leishmania* spp. incriminated as etiological agents of ACL, namely: *Leishmania* (*V*.) *braziliensis* Vianna, 1911, *L*. (*V*.) *guyanensis* Floch, 1954, *L*. (*L*.) *amazonensis* Lainson and Shaw, 1972, *L*. (*V*.) *lainsoni* Silveira et al., 1987, *L*. (*V*.) *shawi* Lainson et al., 1989, *L*. (*V*.) *naiffi* Lainson and Shaw, 1989 and *L*. (*V*.) *lindenbergi* Silveira et al., 2002. All have been well characterized by isoenzyme electrophoresis and the indirect immunofluorescence antibody test (IFAT) using species-specific monoclonal antibodies (McAbs) [21, 48, 49].

The description of most of these leishmanial parasites has been based on strains isolated either from human cutaneous disease (e.g., *L*. (*V*.) *lainsoni* and *L*. (*V*.) *lindenbergi*) or wild reservoir hosts (e.g., *L*. (*L*.) *amazonensis*, *L*. (*V*.) *shawi* and *L*. (*V*.) *naiffi*) in the northeastern and southeastern regions of Pará state, Brazil. Their geographical distribution in other regions of this state is poorly known. Many cases of ACL have been reported in western Pará state but there is very little information on the etiological agents in this area.

The aim of our present study is to phenotypically characterize 43 *Leishmania* spp. isolates from human cases of ACL from western Pará state using isoenzyme electrophoresis (6PGDH, PGM, G6PD, MPI, ASAT, and ALAT) and 23 *Leishmania*-specific monoclonal antibodies (McAbs). These methods have been used for more than 20 years by the Leishmaniasis Research Group of the Instituto Evandro Chagas (IEC) in Pará state, Brazil, in their studies on the taxonomy and eco-epidemiology of leishmanial parasites causing ACL in Amazonian Brazil [23, 25–27, 30, 31, 41–46].

## Material and methods

### Study area

**Figure 1.**
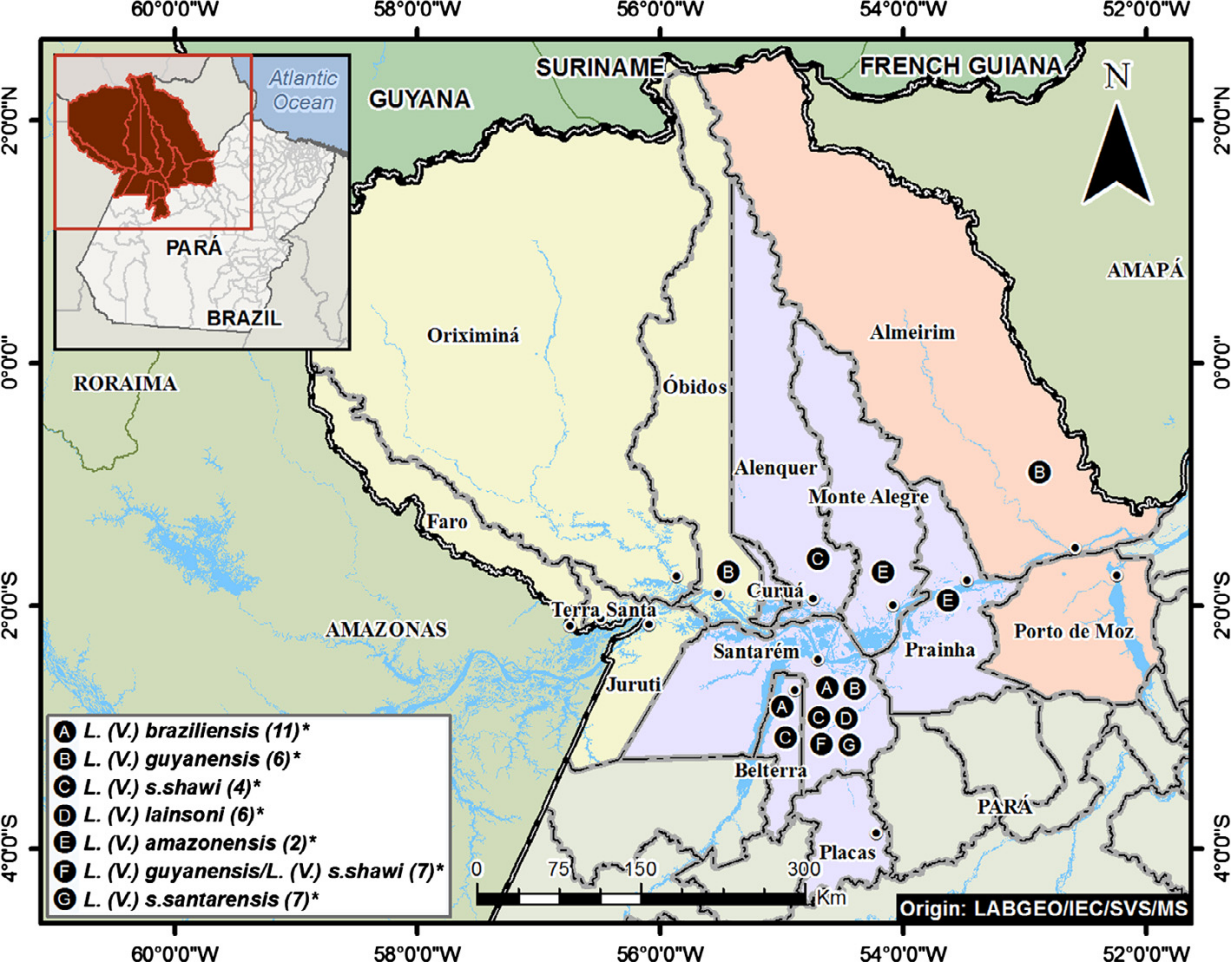
The lower Amazon mesoregion, western Pará state, Brazil, with its three respective microregions (Santarém (lilac), Óbidos (yellow) and Almeirim (orange) and fourteen municipalities, and the distribution and frequency (*) of each *Leishmania* spp. identified in that region.

Our study was carried out in the lower Amazon region of western Pará state, Brazil, that is identified as the lower Amazon mesoregion of Pará by the “Instituto Brasileiro de Geografia e Estatística” [16]. This mesoregion is composed of three microregions: Santarém, Óbidos and Almeirim, and 14 municipalities (Fig. 1, Table 1). These three microregions are among the largest municipalities of Pará state and the population of Santarém is comparable with that of Marabá in southeast Pará and Belém in northeast Pará.

**Table 1.** The fourteen municipalities that compose the three microregions of the lower Amazon mesoregion, western Pará state, Brazil, with their respective geographical area (km^2^) and coordinates.

Municipality	Area (km^2^)	Geographical coordinate
	Micro-region of Óbidos	
Faro	11.820,3	2° 10′ 11″ S: 56° 44′ 32″ W
Juruti	8.342,8	2° 9′ 12″ S: 56° 5′ 14″ W
Óbidos	26.825,5	1° 54′ 7″ S: 55° 31′ 11″ W
Terra Santa	1.909,0	2° 6′ 16″ S: 56° 29′ 15″ W
Oriximiná	108.086,0	1° 45′ 36″ S: 55° 51′ 45″ W
	Micro-region of Santarém	
Alenquer	25.976,9	1° 56′ 33″ S: 54° 44′ 15″ W
Monte Alegre	20.232,5	1° 59′ 56″ S: 54° 4′ 58″ W
Prainha	13.895,7	1° 47′ 39″ S: 53° 28′ 32″ W
Santarém	34.091,0	2° 26′ 22″ S: 54° 41′ 55″ W
Belterra	2.640,6	2° 41′ 54″ S: 54° 53′ 18″ W
Curuá	1.473,6	1° 54′ 19″ S: 55° 10′ 11″ W
Placas	7.194,1	3° 52′ 23″ S: 54° 13′ 11″ W
	Micro-region of Almeirim	
Almeirim	73.287,8	1° 31′ 14″ S: 52° 34′ 53″ W
Porto de Moz	17.500,8	1° 44′ 54″ S: 52° 14′ 18″ W

The mesoregion is a plain containing small hills with maximum altitudes of 100 m. The climate is typically equatorial, with average temperatures ranging from 24 to 26 °C and high humidity. The annual rainfall is approximately 2500 mm, and its rainy season is from January to June. The vegetation consists of an immense rainforest, mainly primary, with inundated areas referred to as *varzeas* and *igapós*.

### *Leishmania* spp. isolated from patients

The 43 isolates of *Leishmania* spp. were obtained from human cases of localized cutaneous leishmaniasis (LCL) [47] examined within two periods; the first one during 1990, 1996, and 1997 when our laboratory collaborated with the Health Secretary of Santarém, to improve their diagnosis of the disease in this municipality. In that period 21 isolates were collected; in 2001 a second batch of another 22 isolates was obtained, during a formal collaboration with that Health Secretary. Of these, 33 were from Santarém, 3 from Belterra, 1 from Prainha (on the southern bank of the Amazon River), 3 from Óbidos, and single isolates from Alenquer, Monte Alegre and Almeirim (on the northern bank of the Amazon River).

### Patients

All patients were examined at the Zoonosis Control Center, Health Secretary, Santarém, and submitted to parasitological diagnosis of the disease as follows:

- For the detection of amastigotes, smears of exudates from the lesions were rapidly air-dried, fixed in absolute methyl alcohol and stained by Giemsa’s method; during 1990, 1996, and 1997, a small volume (≈50 μL) of these exudates was inoculated intradermally into the feet of hamsters for parasite isolation;
- During 2001 triturated tissue from punch biopsies was inoculated into the feet of hamsters and cultivated in Difco B45 culture medium [54].

### Ethical approval

This study was approved by the Ethics Committee in Human Research of the “Núcleo de Medicina Tropical” of the “Universidade Federal do Pará”, Brazil, with the protocol number 22/2000 (ECHR/TMN/FUPa/Brazil). All patients examined within the 2001 period signed an informed consent form. The patients examined during the 1990, 1996 and 1997 periods did not sign the form as they were only submitted to routine proceedings for diagnosis of the disease.

### Phenotypic characterization of *Leishmania* spp. isolated from patients

The phenotypic characterization of *Leishmania* spp. isolated from patients was based on the use of McAbs against the reference strains of *Leishmania* spp. from the Brazilian Amazon Region [15, 41] and on the comparison of the isoenzyme electrophoretic profile and the zymodeme of each isolate with these *Leishmania* spp. [7, 26, 30, 31]:

#### a) *Monoclonal antibodies* (*McAbs*)

Culture forms of each strain were fixed in acetone and tested against a battery of 23 McAbs (B2, B5, B12, B11, B13, B18, B19, CO1, CO2, CO3, D13, L1, LA2, M2, N2, N3, V1, WA2, W1, W2, WH1, WIC.79.3, and T3) produced against different *Leishmania* species [15, 32, 33], using the indirect immunofluorescence/fluorescein-labeled avidin technique [41]. The B and N series react with species of the subgenus *L*. (*Viannia*); D13, M2, T3, WIC.79.3, W1, W2, WA2, and V1 react with parasites of the subgenus *L*. (*Leishmania*); WH1 reacts with parasites of the *L*. *hertigi* complex; LA2 reacts with some strains of *L*. (*V*.) *lainsoni*; CO1, CO2, CO3, and L1 are group-specific and react with members of the genus *Endotrypanum* and some species of *Trypanosoma*.

#### b) *Isoenzyme electrophoresis and zymodeme analysis*

The 6PGDH, PGM, G6PD, MPI, ASAT, and ALAT enzyme profiles of the strains isolated in the present study were prepared according to the methods described by Miles et al. [31]. They were also analyzed according to the position of the electrophoretic bands for the six enzymes. Each electrophoretic band was regarded as a separate character and was numbered from the most distal to the anodic point in each zymogram. Zymodemes were identified according to the pattern of the electrophoretic profiles for the six enzymes [7]. The enzymatic profiles and zymodemes were compared with those of the following reference strains of *Leishmania* species as shown in Table 2. All *Leishmania* reference strains are maintained in the Leishmaniasis Research Group’s cryobank (at −180 °C) located in the IEC’s Parasitology Department, Ananindeua, Pará state, Brazil.

**Table 2.** Enzymatic profiles and zymodemes of the reference strains of *Leishmania* spp. from Amazonian Brazil used by the Leishmaniasis Research Group of the “Instituto Evandro Chagas”, Pará state, Brazil.

		Enzymatic profile					
*Leishmania* spp. (WHO code)	Zymodeme	6PGDH	G6PD	PGM	MPI	ASAT	ALAT
*L*. (*V*.) *braziliensis* (MHOM/BR/75/M2903)	(IEC*-Z1)	3	3	2	5	4	4
*L*. (*V*.) *guyanensis* (MHOM/BR/75/M4147)	(IEC*-Z2)	2	2	3	6	2	2
*L*. (*V*.) *lainsoni* (MHOM/BR/81/M6426)	(IEC*-Z4)	6	5	1	2	2	1
*L*. (*V*.) *s*. *shawi* (MCEB/BR/84/M8408)	(IEC*-Z5)	5	1	4	7	3	2
*L*. (*V*.) *naiffi* (MDAS/BR/79/M5533)	(IEC*-Z6)	4	4	5	3	4	3
*L*. (*L*.) *amazonensis* (IFLA/BR/67/PH8)	(IEC*-Z3)	1	6	6	1	1	5
*L*. (*V*.) *lindenbergi* (MHOM/BR/98/M16714)	(IEC*-Z7)	7	4	5	4	5	3

* IEC = Instituto Evandro Chagas.

## Results

### a) *McAbs*

**Figure 2.**
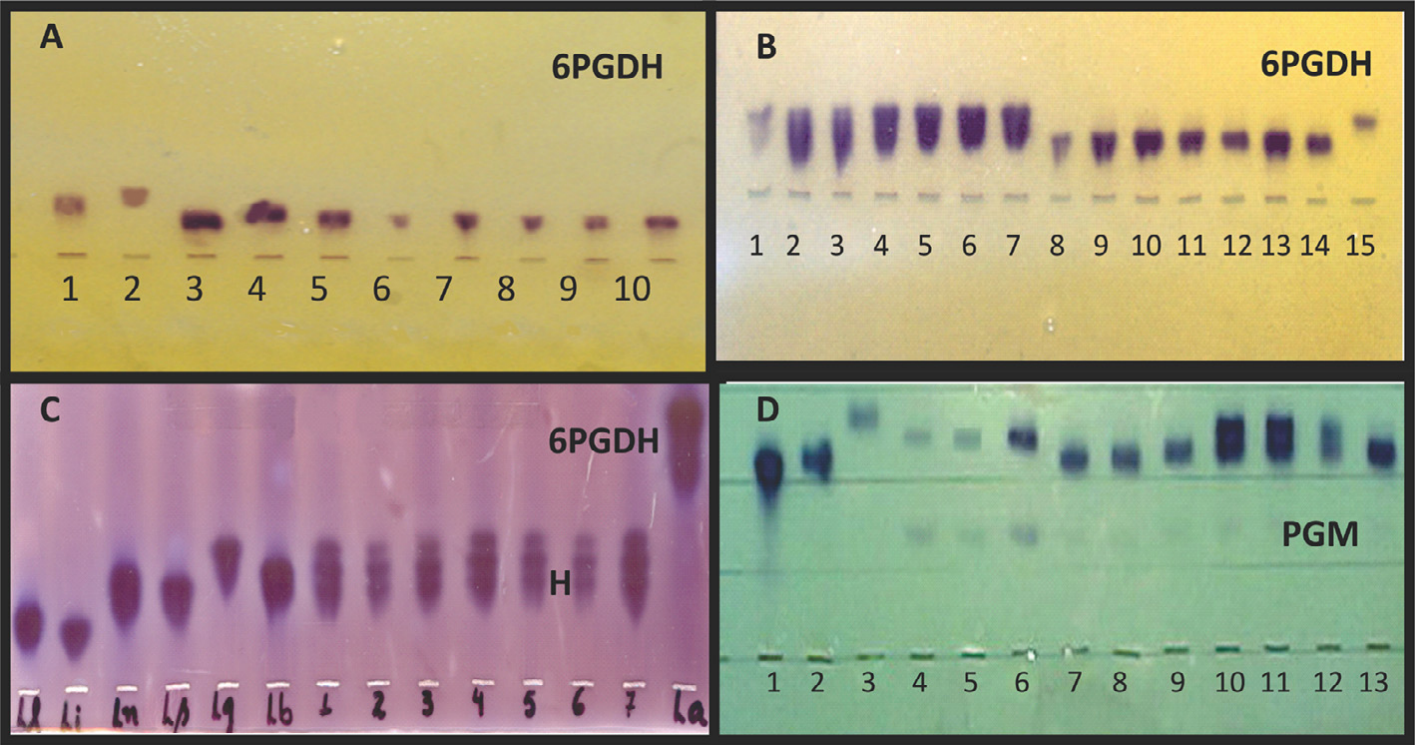
The isoenzyme electrophoresis analysis for identifying *Leishmania* spp. isolated from human cases of cutaneous leishmaniasis from the lower Amazon mesoregion, western Pará state, Brazil. (*A*) (*6PGDH*): The electrophoretic profiles of four isolates of *L*. (*V*.) *s*. *shawi* that did not cross-react with the *L*. (*V*.) *guyanensis* species-specific McAb B19 epitope compared with those of the reference strains of Brazilian Amazon *Leishmania* species. Reading from left to right: 1-*L*. (*V*.) *braziliensis* (MHOM/BR/75/M2903); 2-*L*. (*V*.) *guyanensis* (MHOM/BR/75/M4147); 3-*L*. (*V*.) *lainsoni* (MHOM/BR/81/M6426); 4-*L*. (*V*.) *naiffi* (MDAS/BR/79/M5533); 5 and 10, the reference strain of *L*. (*V*.) *s*. *shawi* (MCEB/BR/84/M8408); 6–9, the four isolates of *L*. (*V*.) *s*. *shawi*: 6-M19664 (MHOM/BR/2001/M19664), 7-M15992 (MHOM/BR/96/M15992), 8-M19670 (MHOM/BR/2001/M19670) and 9-M19703 (MHOM/BR/2001/M19703); (*B*) (*6PGDH*): The electrophoretic profiles of six isolates of *L*. (*V*.) *guyanensis* and seven of *L*. (*V*.) *s*. *santarensis* that cross-reacted with the *L*. (*V*.) *guyanensis* species-specific McAb B19 epitope compared with that of the reference strain of *L*. (*V*.) *guyanensis*. Reading from left to right: 1 and 15, the reference strain of *L*. (*V*.) *guyanensis* (MHOM/BR/75/M4147); 2–7, the six isolates of *L*. (*V*.) *guyanensis*: 2-M19869 (MHOM/BR/2001/M19869), 3-M19663 (MHOM/BR/2001/M19663), 4-M15989 (MHOM/BR/96/M15989), 5-M16174 (MHOM/BR/97/M16174), 6-M13245 (MHOM/BR/90/M13245) and 7-M13102 (MHOM/BR/90/M13102); 8–14, the seven isolates of *L*. (*V*.) *s*. *santarensis*: 8-M19671 (MHOM/BR/2001/M19671), 9-M19693 (MHOM/BR/2001/M19693), 10-M19694 (MHOM/BR/2001/M19694), 11-M15982 (MHOM/BR/96/M15982), 12-M15985 (MHOM/BR/96/M15985), 13-M13070 (MHOM/BR/90/M13070) and 14-M15981 (MHOM/BR/96/M15981); (*C*) (*6PGDH*): The electrophoretic profiles of seven isolates of a putative parasite, *L*. (*V*.) *guyanensis*/*L*. (*V*.) *s*. *shawi*, compared with those of the reference strains of Brazilian Amazon *Leishmania* species. Reading from left to right: *L*.*l*. – *L*. (*V*.) *lainsoni* (MHOM/BR/81/M6426); *L*.*i*. – *L*. (*V*.) *lindenbergi* (MHOM/BR/98/M16714); *L*.*n*. – *L*. (*V*.) *naiffi* (MDAS/BR/79/M5533); *L*.*s*. – *L*. (*V*.) *s*. *shawi* (MCEB/BR/84/M8408); *L*.*g*. – *L*. (*V*.) *guyanensis* (MHOM/BR/75/M4147); *L*.*b*. – *L*. (*V*.) *braziliensis* (MHOM/BR/75/M2903); 1–7, the seven isolates of *L*. (*V*.) *guyanensis*/*L*. (*V*.) *s*. *shawi*: 7-M19672 (MHOM/BR/2001/M19672), 8-M19676 (MHOM/BR/2001/M19676), 9-M15983 (MHOM/BR/96/M15983), 10-M15984 (MHOM/BR/96/M15984), 11-M15987 (MHOM/BR/96/M15987), 12-M15988 (MHOM/BR/96/M15988) and 13-M19697 (MHOM/BR/2001/M19697) and, *L*.*a*. – *L*. (*L*.) *amazonensis* (IFLA/BR/67/PH8); (*D*) (*PGM*): The electrophoretic profiles of seven isolates of a putative parasite, *L*. (*V*.) *guyanensis*/*L*. (*V*.) *s*. *shawi*, compared with those of the reference strains of Brazilian Amazon *Leishmania* species. Reading from left to right: 1-*L*. (*V*.) *naiffi* (MDAS/BR/79/M5533); 2-*L*. (*V*.) *s*. *shawi* (MCEB/BR/84/M8408); 3-*L*. (*V*.) *braziliensis* (MHOM/BR/75/M2903); 4-*L*. (*V*.) *guyanensis* (MHOM/BR/75/M4147) from Pará State; 5-*L*. (*V*.) *guyanensis* (M16343) and 6-*L*. (*V*.) *guyanensis* (M16328) from Amazonas State; 7–13, the seven isolates of *L*. (*V*.) *guyanensis*/*L*. (*V*.) *s*. *shawi*: 7-M19672 (MHOM/BR/2001/M19672), 8-M19676 (MHOM/BR/2001/M19676), 9-M15983 (MHOM/BR/96/M15983), 10-M15984 (MHOM/BR/96/M15984), 11-M15987 (MHOM/BR/96/M15987), 12-M15988 (MHOM/BR/96/M15988) and 13-M19697 (MHOM/BR/2001/M19697). Scale: the distance between the points of origin of each parasite is approximately 1.0 cm.

Twenty-six of the 43 isolates were preliminarily identified with McAbs as follows: *L*. (*V*.) *braziliensis* (11/25.6%), *L*. (*V*.) *guyanensis* (13/30.2%), and *L*. (*L*.) *amazonensis* (2/4.6%). However, some of these preliminary identifications were not confirmed by the isoenzyme electrophoresis and zymodeme analysis. We identified seven isolates as *L*. (*V*.) *guyanensis* because of their positive reaction with the McAb B19, but their enzymatic profiles were identical to that of the *L*. (*V*.) *shawi* reference strain (Fig. 2B, Table 3).

**Table 3.** McAb reaction profiles and serodemes, enzymatic profiles and zymodemes of 43 strains of *Leishmania* spp. isolated from human cases of cutaneous leishmaniasis from the lower Amazon mesoregion, western Pará state, Brazil.

*Leishmania* spp. WHO code	McAb reaction-profile	Serodeme	Species^a^	Enzymatic profile^b^	Zymodeme	Locality^c^
MHOM/BR/2001/M19678	B2, B5, B12, B18, L1, N2	I	*L*. (*V*.) *braziliensis*	3; 3; 2; 5; 4; 4	IEC-Z1	1
MHOM/BR/2001/M19698	B2, B5, B12, B18, L1, N2	I	*L*. (*V*.) *braziliensis*	3; 3; 2; 5; 4; 4	IEC-Z1	1
MHOM/BR/2001/M19669	B2, B5, B12, B18, L1, N2	I	*L*. (*V*.) *braziliensis*	3; 3; 2; 5; 4; 4	IEC-Z1	1
MHOM/BR/2001/M19665	B2, B5, B12, B18, L1, N2	I	*L*. (*V*.) *braziliensis*	3; 3; 2; 5; 4; 4	IEC-Z1	5
MHOM/BR/2001/M19667	B2, B5, B12, B18, L1, N2	I	*L*. (*V*.) *braziliensis*	3; 3; 2; 5; 4; 4	IEC-Z1	1
MHOM/BR/2001/M19673	B2, B5, B12, B18, L1, N2	I	*L*. (*V*.) *braziliensis*	3; 3; 2; 5; 4; 4	IEC-Z1	1
MHOM/BR/1996/M15991	B2, B5, B12, B18, L1, N2	I	*L*. (*V*.) *braziliensis*	3; 3; 2; 5; 4; 4	IEC-Z1	1
MHOM/BR/1996/M15986	B2, B12, B18, L1, N2	II	*L*. (*V*.) *braziliensis*	3; 3; 2; 5; 4; 4	IEC-Z1	1
MHOM/BR/1996/M15923	B5, B12, B18, L1, N2	III	*L*. (*V*.) *braziliensis*	3; 3; 2; 5; 4; 4	IEC-Z1	1
MHOM/BR/1996/M15866	B5, B12, B18, L1, N2	III	*L*. (*V*.) *braziliensis*	3; 3; 2; 5; 4; 4	IEC-Z1	1
MHOM/BR/1996/M15990	B5, B12, B18, L1, N2	III	*L*. (*V*.) *braziliensis*	3; 3; 2; 5; 4; 4	IEC-Z1	1
MHOM/BR/2001/M19869	B2, B12V, B19, L1 – II	–	*L*. (*V*.) *guyanensis*	2; 2; 3; 6; 2; 2	IEC-Z2	8
MHOM/BR/1997/M16174	B2, B12V, B19, L1 – II	–	*L*. (*V*.) *guyanensis*	2; 2; 3; 6; 2; 2	IEC-Z2	8
MHOM/BR/1990/M13245	B2, B12V, B19, L1 – II	–	*L*. (*V*.) *guyanensis*	2; 2; 3; 6; 2; 2	IEC-Z2	8
MHOM/BR/1990/M13102	B2, B12V, B19, L1 – II	–	*L*. (*V*.) *guyanensis*	2; 2; 3; 6; 2; 2	IEC-Z2	13
MHOM/BR/2001/M19663	B2, B12V, B19, L1 – II	–	*L*. (*V*.) *guyanensis*	2; 2; 3; 6; 2; 2	IEC-Z2	1
MHOM/BR/2001/M19671*	B2, B12V, B19, L1 – II	–	*L*. (*V*.)*s*. *santarensis*	5; 1; 4; 7; 3; 2	IEC-Z5	1
MHOM/BR/2001/M19693*	B2, B12V, B19, L1 – II	–	*L*. (*V*.) *s*. *santarensis*	5; 1; 4; 7; 3; 2	IEC-Z5	1
MHOM/BR/2001/M19694*	B2, B12V, B19, L1 – II	–	*L*. (*V*.) *s*. *santarensis*	5; 1; 4; 7; 3; 2	IEC-Z5	1
MHOM/BR/1996/M15982*	B2, B12V, B19, L1 – II	–	*L*. (*V*.) *s*. *santarensis*	5; 1; 4; 7; 3; 2	IEC-Z5	1
MHOM/BR/1996/M15989	B2, B12V, B19, L1 – II	–	*L*. (*V*.) *guyanensis*	2; 2; 3; 6; 2; 2	IEC-Z2	1
MHOM/BR/1996/M15985*	B2, B12V, B19, L1 – II	–	*L*. (*V*.) *s*. *santarensis*	5; 1; 4; 7; 3; 2	IEC-Z5	1
MHOM/BR/1990/M13070*	B2, B12V, B19, L1 – II	–	*L*. (*V*.) *s*. *santarensis*	5; 1; 4; 7; 3; 2	IEC-Z5	1
MHOM/BR/1996/M15981*	B2, B12V, B19, L1 – II	–	*L*. (*V*.) *s*. *santarensis*	5; 1; 4; 7; 3; 2	IEC-Z5	1
MHOM/BR/1996/M15996	M2, W1, WA2V, L1 – III	–	*L*. (*L*.) *amazonensis*	1; 6; 6; 1; 1; 5	IEC-Z3	2
MHOM/BR/1996/M15807	M2, W1, WA2V, L1 – III	–	*L*. (*L*.) *amazonensis*	1; 6; 6; 1; 1; 5	IEC-Z3	4
MHOM/BR/2001/M19664	B2, B12, L1 – IV	–	*L*. (*V*.) *s*. *shawi*	5; 1; 4; 7; 3; 2	IEC-Z5	3
MHOM/BR/1996/M15992	B2, B12, L1 – IV	–	*L*. (*V*.) *s*. *shawi*	5; 1; 4; 7; 3; 2	IEC-Z5	1
MHOM/BR/2001/M19670	B2, B12, L1 – IV	–	*L*. (*V*.) *s*. *shawi*	5; 1; 4; 7; 3; 2	IEC-Z5	5
MHOM/BR/1990/M19703	B2, B12, L1 – IV	–	*L*. (*V*.) *s*. *shawi*	5; 1; 4; 7; 3; 2	IEC-Z5	5
MHOM/BR/2001/M19675	L1 – V	–	*L*. (*V*.) *lainsoni*	6; 5; 1; 2; 2; 1	IEC-Z4	1
MHOM/BR/2001/M19690	L1 – V	–	*L*. (*V*.) *lainsoni*	6; 5; 1; 2; 2; 1	IEC-Z4	1
MHOM/BR/2001/M19692	L1 – V	–	*L*. (*V*.) *lainsoni*	6; 5; 1; 2; 2; 1	IEC-Z4	1
MHOM/BR/2001/M19696	L1 – V	–	*L*. (*V*.) *lainsoni*	6; 5; 1; 2; 2; 1	IEC-Z4	1
MHOM/BR/2001/M19701	L1 – V	–	*L*. (*V*.) *lainsoni*	6; 5; 1; 2; 2; 1	IEC-Z4	1
MHOM/BR/2001/M19702	L1 – V	–	*L*. (*V*.) *lainsoni*	6; 5; 1; 2; 2; 1	IEC-Z4	1
MHOM/BR/2001/M19672	B2, B12V, L1 – VI	–	*L*. (*V*.) *guyanensis/*	2,3,5; 2; 4; 6;	IEC-Z9	1
			*L*. (*V*.) *s*. *shawi*/var2	5; 2		
MHOM/BR/2001/M19676	B2, B12V, L1 – VI	–	*L*. (*V*.) *guyanensis/*	2,3,5; 2; 4; 6;	IEC-Z9	1
			*L*. (*V*.) *s*. *shawi*/var2	5; 2		
MHOM/BR/2001/M19697	B2, B12V, L1 – VI	–	*L*. (*V*.) *guyanensis/*	2,3,5; 2; 4; 6;	IEC-Z9	1
			*L*. (*V*.) *s*. *shawi*/var2	5; 2		
MHOM/BR/1996/M15983	B2, B12V, L1 – VI	–	*L*. (*V*.) *guyanensis/*	2,3,5; 2; 4; 6;	IEC-Z9	1
			*L*. (*V*.) *s*. *shawi*/var2	5; 2		
MHOM/BR/1996/M15984	B2, B12V, L1 – VI	–	*L*. (*V*.) *guyanensis/*	2,3,5; 2; 3; 6;	IEC-Z8	1
			*L*. (*V*.) *s*. *shawi*/var1	5; 2		
MHOM/BR/1996/M15987	B2, B12V, L1 – VI	–	*L*. (*V*.) *guyanensis/*	2,3,5; 2; 3; 6;	IEC-Z8	1
			*L*. (*V*.) *s*. *shawi*/var1	5; 2		
MHOM/BR/1996/M15988	B2, B12V, L1 – VI	–	*L*. (*V*.) *guyanensis/*	2,3,5; 2; 3; 6;	IEC-Z8	1
			*L*. (*V*.) *s*. *shawi*/var1	5; 2		

a Definitive nomination of *Leishmania* spp.

b Order of enzymes used for characterization of enzymatic profiles: 6PGDH, G6PD, PGM, MPI, ASAT, ALAT.

c Santarém^(1)^, Monte Alegre^(2)^, Alenquer^(3)^, Prainha^(4)^, Belterra^(5)^, Curuá^(6)^, Placas^(7)^, Óbidos^(8)^, Faro^(9)^, Juruti^(10)^, Terra Santa^(11)^, Oriximiná^(12)^, Almeirim^(13)^ and Porto de Moz^(14)^.

* Strains of *L*. (*V*.) *s*. *santarensis* that cross-reacted to McAb B19 epitope and were later identified by their isoenzyme electrophoretic profile.

#### *L.* (*V*.) *braziliensis*

Eleven (25.6%) isolates reacted with the McAbs B18, B2, B5, B12, N2, and L1, identifying them as *L*. (*V*.) *braziliensis*. The 11 strains were classified into 3 serodemes: S – I (7 isolates), recognized by the McAbs B2, B5, B12, B18, N2, and L1; S – II (one isolate) that did not express the B5 epitope; S – III (3 isolates) that did not express the B2 epitope (Table 3).

#### *L.* (*V*.) *guyanensis*

Thirteen (30.2%) isolates reacted with the McAb B19, which is considered to be specific for *L*. (*V*.) *guyanensis*, and the group-specific McAbs B2, B12V, and L1, characterizing them as McAb profile II. However, their enzymatic profile only confirmed 6 of these as being *L*. (*V*.) *guyanensis* (Table 3).

#### *L.* (*L*.) *amazonensis*

Two (25.6%) isolates of *L*. (*L*.) *amazonensis* were identified by their reaction with the McAbs M2, W1, WA2V, and L1 epitopes, characterizing them as McAb profile III (Table 3).

### *Leishmania* spp. with non-specific McAb reaction profiles

Seventeen isolates did not react with the McAbs B18 and B19, which are considered to be specific for *L*. (*V*.) *braziliensis* and *L*. (*V*.) *guyanensis*, and we grouped them as belonging to McAb profiles IV, V, and VI (Table 3).

Of these, 4 isolates reacted with the group-specific McAbs B2, B12, and L1, indicating they belonged to the *Viannia* subgenus and characterized as McAb profile IV.

A further 6 isolates reacted with McAb L1, that were characterized as McAb profile V.

The McAb profile of 7 other isolates was similar to profile IV but a variable number of parasites expressed the B12 epitope, which is typical of *L*. (*V*.) *guyanensis*, and was designated as profile VI.

### b) *Isoenzyme electrophoresis and zymodeme analysis*

Based on the isoenzyme electrophoresis and zymodeme analysis, we identified the 43 strains as follows: 11 (25.6%) as *L*. (*V*.) *braziliensis* – 10 from Santarém and 1 from Belterra; 6 (13.9%) as *L*. (*V*.) *lainsoni* – from Santarém municipality; 11 (25.6%) as *L*. (*V*.) *shawi*: 1 from Alenquer, 8 from Santarém and 2 from Belterra; 6 (13.9%) as *L*. (*V*.) *guyanensis* – 2 from Santarém, 3 from Óbidos and 1 from Almeirim, with only 33.3% from the southern bank of the Amazon River; 2 (4.7%) as *L*. (*L*.) *amazonensis*, with 1 from the municipality of Prainha and the other from that of Monte Alegre municipality; and 7 (16.3%) as putative hybrid *Leishmania* (*V*.) *guyanensis*/*Leishmania* (*V*.) *shawi shawi* parasites from Santarém municipality (Table 3).

#### *L*. (*V*.) *braziliensis*

The electrophoretic mobility of the 6PGDH, MPI, G6PD, PGM, ASAT, and ALAT enzymes of 11 isolates was identical to that of the *L*. (*V*.) *braziliensis* reference strain, configuring the zymodeme IEC-Z1 (Tables 2 and 3). This confirmed their previous identification by the species-specific McAb B18 epitope. In addition, it is important to note that there was no enzymatic polymorphism among these isolates.

#### *L*. (*V*.) *guyanensis*

The electrophoretic mobility of the 6PGDH, MPI, G6PD, PGM, ASAT, and ALAT enzymes of 6 isolates was identical to that of the *L*. (*V*.) *guyanensis* reference strain, configuring the zymodeme IEC-Z2 (Tables 2 and 3, Fig. 2B).

#### *L*. (*L*.) *amazonensis*

The electrophoretic mobility of the 6PGDH, MPI, G6PD, PGM, ASAT, and ALAT enzymes of 2 isolates was identical to that of the *L*. (*L*.) *amazonensis* reference strain, confirming the zymodeme IEC-Z3 (Tables 2 and 3).

#### *L*. (*V*.) *lainsoni*

The electrophoretic mobility of the 6PGDH, MPI, G6PD, PGM, ASAT, and ALAT enzymes of 6 isolates was identical to that of the *L*. (*V*.) *lainsoni* reference strain, confirming the zymodeme IEC-Z4 (Tables 2 and 3).

#### *L*. (*V*.) *shawi shawi* and *L*. (*V*.) *shawi santarensis*

The electrophoretic mobility of the 6PGDH, MPI, G6PD, PGM, ASAT, and ALAT enzymes of 11 strains was identical to that of the *L*. (*V*.) *shawi* reference strain (Figs. 2A and 2B), corroborating the zymodeme IEC-Z5 (Tables 2 and 3). However, based on their McAb reaction profiles, these could be divided into two populations. One was represented by four isolates that did not express the B19 epitope, while the other one was composed of 7 isolates that expressed the B19 epitope. This is the first record of a population of *L*. (*V*.) *shawi* expressing the B19 epitope. Based on these observations, we have given the two populations of *L*. (*V*.) *shawi* sub-specific rank as follows: *Leishmania* (*V*.) *shawi shawi* for the eastern population of the reference strain and the four isolates from the western population with the McAb reaction profile like the type species reacting with the McAbs B2, B12, and L1 (McAb reaction profile IV), and *L*. (*V*.) *shawi santarensis* for the western population reacting with the McAbs B2, B12V, B19, and L1 (McAb reaction profile II). The four strains of *L*. (*V*.) *s*. *shawi* from the western region are from Alenquer, Santarém and two from Belterra, and all seven of *L*. (*V*.) *s*. *santarensis* are from the Santarém municipality (Table 3). Thus, 91% are from the southern bank of the Amazon River.

#### *L*. (*V*.) *guyanensis/L*. (*V*.) *shawi shawi*, a putative hybrid parasite from *L*. (*V*.) *guyanensis* and *L*. (*V*.) *shawi shawi*

The electrophoretic mobility of the 6PGDH, MPI, G6PD, PGM, ASAT, and ALAT enzymes of 7 isolates confirmed them as belonging to the subgenus *Viannia* but their 6PGDH profile had three bands at the same level as the single band of the *L*. (*V*.) *guyanensis* reference strain. However, their PGM profile varied, being more similar to that of the *L*. (*V*.) *s*. *shawi* reference strain (Figs. 2C and 2D). This leads us to suggest that these two new enzymatic profiles with their respective zymodemes, IEC-Z8 and IEC-Z9, represent putative hybrid parasites of *L*. (*V*.) *guyanensis* and *L*. (*V*.) *shawi shawi* (Table 3).

## Discussion

Although a high occurrence of ACL is well known in the lower Amazon mesoregion of Pará state, there is very little information on the etiological agents of the disease in this area. This region is ecologically very interesting, being at a point where there was a land link between the northern and southern banks of the Amazon River, which is now an important biological barrier for some animal species.

In this study we identified four of the seven different *Leishmania* species that are incriminated as the etiological agents of ACL in Pará state, namely, *L*. (*V*.) *braziliensis* (11/25.6%), *L*. (*V*.) *guyanensis* (6/13.9%), *L*. (*V*.) *lainsoni* (6/13.,9%), *L*. (*L*.) *amazonensis* (2/4.7%), and two sub-species of *L*. (*V*.) *shawi*. One is *L*. (*V*.) *shawi shawi* (4/9.3%) and the other we have designated as a new sub-species *L*. (*V*.) *shawi santarensis* (7/16.3%). We also identified, for the first time in this region, a putative hybrid leishmanial parasite, *L*. (*V*.) *guyanensis/L*. (*V*.) *s*. *shawi* (7/16.3%), causing ACL in Amazonian Brazil. However, we failed to indicate the presence of *L*. (*V*.) *naiffi* and *L*. (*V*.) *lindenbergi*.

The low prevalence of ACL due to *L*. (*V*.) *naiffi* in Pará state, Brazil [4] may be due to a number of epidemiological factors [21]. In the first place, and unlike most neotropical species of *Leishmania*, this parasite frequently produces no visible lesion in the skin of the hamster, although it may be re-isolated following the in vitro culture of triturated tissue from the site of inoculation. If similar occult infections are produced in some individuals the infection rate of *L*. (*V*.) *naiffi* in man may be much higher than previously thought. Secondly, the sand fly *Psychodopygus ayrozai* Barreto and Coutinho, 1940 is generally considered as the vector transmitting the parasite among the reservoir hosts, the armadillo *Dasipus novemcinctus*. This insect has been found naturally infected by *L*. (*V*.) *naiffi* [45] and is a very frequent occupant of armadillo burrows. In the Amazonian forest, however, *Ps*. *ayrozai* is not very anthropophilic, resulting in only sporadic transmission of *L*. (*V*.) *naiffi* to man by this species of sand fly. Only very rare infections have been recorded in the two highly anthropophilic sand flies, *Ps*. *paraensis* Costa Lima, 1941 and *Ps*. *squamiventris* Lutz and Neiva, 1912, in spite of the large number of these two insects dissected during studies on the epidemiology of ACL, and this suggests that they are not very attracted to armadillos.

*L*. (*V*.) *lindenbergi*, for unknown reasons, still seems to be restricted to the type locality in the municipality of Belém, Pará state [46] and contiguous municipalities such as Ananindeua, Marituba, Benevides, Santa Bárbara, and Santa Isabel [38].

The first readily distinguishable *Leishmania* species identified in this study was *L*. (*V*.) *braziliensis*, found in 25.6% of the isolates, and all reacted with the McAb B18, which is considered to be specific for this parasite, although a Colombian population of *L*. (*V*.) *braziliensis* did not express this epitope [39]. All the isolates were from the southern bank of the Amazon River, 10 from Santarém and 1 from Belterra. This high frequency confirms the importance of this parasite as an etiological agent of ACL in the lower Amazon mesoregion of Pará state.

Although isoenzyme analysis did not reveal any genetic heterogeneity, we identified three serodemes of *L*. (*V*.) *braziliensis* that have previously been recorded [40] in other regions of Amazonian Brazil. This finding confirmed the occurrence of intra-specific McAb variation of *L*. (*V*.) *braziliensis* with the dominance of the serodeme I (7) followed by serodemes II (1) and III (3).

The zymodeme of our 11 *L*. (*V*.) *braziliensis* isolates was identical to that of the *L*. (*V*.) *braziliensis* reference strain, indicating the presence of a homogeneous population lacking any enzymatic polymorphism in our study area. A number of enzymatic studies in other regions have led to the opinion that *L*. (*V*.) *braziliensis* is polymorphic [5, 10, 35, 36, 53]. The lack of polymorphism in our study is perhaps due to the fact that only three species of phlebotomine sand flies have been incriminated as the vectors of *L*. (*V*.) *braziliensis* in Pará state; the two closely related species *Ps*. *wellcomei* Fraiha, Shaw and Lainson, 1971 and *Ps*. *complexus* Mangabeira Filho, 1941, and *Ps*. *davisi* Root, 1934 [27, 51, 52]. The reservoirs of this parasite have so far not been identified in this region and the vector/reservoir contact may favor genetic stability rather than genetic variability. The crucial role in the identification of *L*. (*V*.) *braziliensis* of the enzymes 6PGDH and PGM must be emphasized, especially that of the former, which is considered the best enzymatic marker for the characterization of *Leishmania* species [2, 8, 13, 17, 19, 37].

The second most frequent *Leishmania* in our study was *L*. (*V*.) *shawi*. This species was originally described in 1989 [28] from parasites isolated from wild animals captured in the Carajás mountain, in the southeast region of Pará state. The type material of this species does not express the B19 epitope and its McAb profile is more similar to that of *L*. (*V*.) *braziliensis* than *L*. (*V*.) *guyanensis*. *L*. (*V*.) *shawi* has also been found in Pernambuco state, in the northeast region of Brazil [3], as well as a putative hybrid of this species with *L*. (*V*.) *braziliensis*. In the absence of a species-specific monoclonal antibody the identification of *L*. (*V*.) *shawi* depends on isoenzyme profiles. With this technique we identified 11 isolates as being *L*. (*V*.) *shawi*, but initially we had identified 7 of these as *L*. (*V*.) *guyanensis* because of their reaction with the McAb B19 and a McAb profile (IV) that is identical to *L*. (*V*.) *guyanensis*. The McAb profile of the other 4 isolates was identical to that of the *L*. (*V*.) *shawi* reference strain, which is closer to that of *L*. (*V*.) *braziliensis* than *L*. (*V*.) *guyanensis*. This clearly showed the presence of two populations of *L*. (*V*.) *shawi*, and based on this we decided to name the one that corresponds to the type species *L*. (*V*.) *shawi shawi* and the other, whose McAb profile is the same as that of *L*. (*V*.) *guyanensis*, *L*. (*V*.) *shawi santarensis*.

Our experience with the *L*. (*V*.) *s*. *shawi* isolates shows that isoenzyme electrophoresis and zymodeme analysis is crucial for identifying *Leishmania*, but that McAb profiles are useful in studying populations of parasites that have identical isoenzyme profiles. So far there is no information as to the functional importance of serodemes within a species but they may reflect different selective pressures related to vectors. This is in keeping with the accepted fact that *Leishmania*-specific monoclonal antibodies have greater discriminatory power than enzyme profiles [6].

The enzyme electrophoretic profile as well as the zymodeme of 6 isolates that expressed the B19 epitope were identical to those of the *L*. (*V*.) *guyanensis* reference strain, indicating the presence of a homogeneous *L*. (*V*.) *guyanensis* population in our study area, even though they were from both sides of the Amazon River. Of these isolates, 33.3% came from the southern bank of the Amazon River (two from Santarém), while the majority (66.7%) were from the northern bank (three from Óbidos and one from Almeirim). This is the first record of *L*. (*V*.) *guyanensis* ACL from the southern bank of the Amazon River. Our present data supports previous observations in which *L*. (*V*.) *guyanensis* is distributed principally east to west along the northern bank of the Amazon River, and *L*. (*V*.) *s*. *shawi* along the southern bank [20, 21].

Five serodemes have been described for *L*. (*V*.) *guyanensis* parasites that express the B19 epitope and these populations are found in the northwestern areas of Pará and Amapá states [40]. However, other serodemes of *L*. (*V*.) *guyanensis*, from the municipality of Manaus, Amazonas state, have been found that do not express the B19 epitope [12, 37] and in Colombia [39]. These serodemes were not found in the present study.

Unlike other members of the subgenus *Viannia*, *L*. (*V*.) *lainsoni* is the only species that does not react with any McAbs that are typical for the subgenus, reacting with just the general trypanosomatid McAb L1 [18, 41]. Recent studies have suggested that some but not all *L*. (*V*.) *lainsoni* isolates may express the LA2 epitope [15, 46]. The isoenzyme electrophoresis and zymodeme analysis confirmed that the six isolates that reacted only with McAb L1 were all *L*. (*V*.) *lainsoni*, and that there was no enzymatic polymorphism among them. All were from Santarém municipality on the southern bank of the Amazon River, confirming the importance of this species in the epidemiology of ACL in the lower Amazon mesoregion, western Pará state, Brazil.

Based on analysis of the McAbs results, only two isolations, from the municipalities of Prainha and Monte Alegre, were identified as *L*. (*L*.) *amazonensis*, results that were confirmed by the isoenzyme electrophoresis and zymodeme analysis. The profiles of both isolates were the same as that of the reference strain. This lack of enzymatic polymorphism agrees with previous studies in the eastern lower Amazon region [25, 29–31]. However, differences in 6PGDH and G6PD profiles found in Amazonian Brazil [50], French Guiana [11], and Colombia [53] show that there is enzyme polymorphism of this species in the Amazon Region.

The MPI, G6PD, ASAT, and ALAT profiles of seven isolates with non-specific McAbs reaction profiles were similar to those of *L*. (*V*.) *guyanensis*. However, the 6PGDH profile of these same isolates had three bands instead of one at the same level as the *L*. (*V*.) *guyanensis* reference strain. On the other hand, their PGM profile was more similar to that of the reference strain of *L*. (*V*.) *s*. *shawi* than *L*. (*V*.) *guyanensis*. These new isoenzyme profiles with their respective zymodemes, IEC-Z8 and IEC-Z9, suggest that these seven isolates represent a putative hybrid population, *L*. (*V*.) *guyanensis*/*L*. (*V*.) *s*. *shawi*. The role of this putative hybrid parasite in the phylogeny of *L*. (*V*.) *guyanensis* and *L*. (*V*.) *s*. *shawi* is uncertain. However, this area represents an interface between north and southern Amazonia, and based on this we speculate that this lower Amazon mesoregion of Pará state can be regarded as a “hybrid zone” where genetic exchange may occur between closely related parasites, such as *L*. (*V*.) *guyanensis* and *L*. (*V*.) *s*. *shawi*, that share the same vectors. It should be highlighted that in the neighboring state of Amapá (Fig. 1), north of Brazil, more exactly in the locality named “Serra do Navio”, on the northern bank of the Amazon River, we have already found the sand fly *Nyssomyia whitmani* Antunes and Coutinho, 1939, the Amazonian recognized vector of *L*. (*V*.) *s*. *shawi* [21, 24], naturally infected by *L*. (*V*.) *guyanensis* (Souza et al. unpublished data).

This is the second report of a putative *Leishmania* hybrid from Amazonian Brazil, the first being a suspected hybrid between *L*. (*V*.) *lainsoni* and *L*. (*V*.) *naiffi* from the state of Acre, in western Brazilian Amazon [9]. However, in contrast to the present finding regarding the putative hybrid parasite, *L*. (*V*.) *guyanensis*/*L*. (*V*.) *s*. *shawi*, derived from the closely related parasites *L*. (*V*.) *guyanensis* and *L*. (*V*.) *s*. *shawi*, a recent phylogenetic analysis of *Leishmania* (*Viannia*) parasites considered *L*. (*V*.) *lainsoni* and *L*. (*V*.) *naiffi* as the most divergent species [1], which suggests that the putative hybrid *L*. (*V*.) *lainsoni*/*L*. (*V*.) *naiffi* seems only to be a fortuitous finding from where *L*. (*V*.) *naiffi* has never been recorded (state of Acre). In addition, molecular analysis (PCR-RFLP ITS1rDNA) of the putative hybrid *L*. (*V*.) *lainsoni*/*L*. (*V*.) *naiffi* confirmed it to be *L*. (*V*.) *lainsoni* [9]. Thus, in spite of these comments we are able to regard *L*. (*V*.) *guyanensis*/*L*. (*V*.) *s*. *shawi* as the first putative *Leishmania* hybrid in the eastern Brazilian Amazon.

Our study shows the importance of using different methods to characterize *Leishmania* and indicates the divergence of characters at different genetic levels. For instance, in spite of high levels of enzymatic homogeneity, we found high levels of epitope variability with monoclonal antibodies. We were surprised to find that the monoclonal antibody profile of one of the *L*. (*V*.) *shawi* populations is identical to that of *L*. (*V*.) *guyanensis*, while that of the other is more similar to that of *L*. (*V*.) *braziliensis*. This leads us to suggest that the former could be due to genetic exchange at some period between *L*. (*V*.) *guyanensis* and *L*. (*V*.) *shawi* when the two populations reencountered each other in this unique bridging zone between northern and southern Amazonia.

In discussing the origin of Amazonian species, Haffer [14] drew attention to the clear separation of closely related avian species north and south of the Amazon River. He also emphasized that species are continually being separated and later reconnected due to climatic and paleogeological changes. The area between Manaus, Amazonas state, and Óbidos, Pará state, was joined for long periods during the Tertiary period and possibly later and allowed the exchange of fauna between the Guinian Shield and the southern Brazilian Shield. Up until the late Miocene this “bridge” was open and separated the upper and lower Amazon Basins [34]. Such a time scale is well within the proposed phylogenies of the American *Leishmania*.

Our finding of putative hybrids between *L*. (*V*.) *guyanensis* and *L*. (*V*.) *s*. *shawi* and sub-speciation of *L*. (*V*.) *shawi* in this area adds more weight to the importance of this region as an ecological bridging zone between northern and southern Amazonia, as has previously been suggested.
